# Vision-language models for zero-shot weed detection and visual reasoning in UAV-based precision agriculture

**DOI:** 10.3389/fpls.2025.1735096

**Published:** 2026-01-29

**Authors:** Muhammad Fahad Nasir, Mobeen Ur Rehman, Irfan Hussain

**Affiliations:** 1 Khalifa University Center for Autonomous Robotic Systems, Khalifa University, Abu Dhabi, United Arab Emirates; 2 College of Information Technology, United Arab Emirates University, Al-Ain, Abu Dhabi, United Arab Emirates

**Keywords:** error-probing prompting, interpretability, multimodal AI, precision agriculture, UAV imagery, vision–language models, weed detection, zero-shot learning

## Abstract

Weeds remain a major constraint to row-crop productivity, yet current deep learning approaches for UAV imagery often require extensive annotation, generalize poorly across fields, and provide limited interpretability. We investigate whether modern vision–language models (VLMs) can address these gaps in a zero-shot setting. Using drone images from soybean fields with ground-truth weed boxes, we evaluate six commercial VLMs, ChatGPT-4.1, ChatGPT-4o, Gemini Flash 2.5, Gemini Flash Lite 2.5, LLaMA-4 Scout, and LLaMA-4 Maverick under a unified prompt that elicits (i) weed presence, (ii) spatial localization, (iii) reasoning, (iv) crop growth stage, and (v) crop type. We further introduce Error-Probing Prompting (EPP), a counterfactual follow-up that forces re-analysis under the assumption that weeds are present, and we quantify self-correction with expert-rated interpretability scores (Grounding, Specificity, Plausibility, Non-Hallucination, Actionability). Across models, Gemini Flash 2.5 delivers the most consistent zero-shot performance and highest interpretability, ChatGPT-4.1 provides the strongest reasoning but lower raw detection, ChatGPT-4o offers a balanced profile, and LLaMA-4 variants lag in localization and specificity. Gemini Flash Lite 2.5 is efficient but fails EPP stress tests, revealing brittle reasoning. Visual grounding analysis and a text-to-region overlap metric show that interpretability tracks spatial correctness. Results highlight that explainability and feedback driven adaptability not scale alone best predict reliability for field deployment, and position VLMs as promising, low-annotation tools for precision weed management.

## 1 Introduction

The rapid spread of weeds remains a persistent obstacle to achieving sustainable crop production worldwide. It reduces yields, increases dependence on herbicides, and contributes to growing environmental concerns. In row-crops such as soybeans, weeds compete for light, nutrients, water, and space, often causing substantial decreases in both biomass and harvestable yield (Liu et al., 2024; Russo et al., 2024; Wang et al., 2019). At the same time, the rise of unmanned aerial vehicles (UAVs) combined with high-resolution imaging has brought a new dimension to precision agriculture. These UAVs can quickly capture detailed information across entire fields, allowing farmers to monitor crops closely and make timely, targeted interventions for managing weeds, pests, and overall crop health (Toscano et al., 2024; Wang et al., 2025a).

With recent progress in imaging technologies, numerous deep learning approaches have been introduced to automate weed detection and segmentation tasks (Rai et al., 2024; Guo et al., 2025; Rehman et al., 2024). Despite their success, these methods usually rely on large, carefully annotated datasets and are often tailored to specific crop-weed combinations, which limits their adaptability under different conditions. Their performance can drop when faced with variations in growth stages, lighting, sensor characteristics, or regional environments. Variability in crop appearance and biological traits further increases visual complexity in agricultural imagery, motivating the need for adaptable perception models Yang et al. (2021); Alharam et al. (2021); Nasir et al. (2025). Moreover, most of these models operate as “black boxes” offering little insight into their decision making processes a significant drawback for real-world agricultural applications, where transparency is crucial (Shams et al., 2024). These challenges underscore the necessity for more flexible, interpretable, and context-aware AI frameworks capable of maintaining robust performance across heterogeneous agricultural settings with minimal human supervision.

Recent advances in multimodal artificial intelligence offer a compelling response to these challenges. Specifically, Vision-Language Models (VLMs) which are trained on massive datasets of images and their corresponding text have recently shown an impressive ability to handle complex visual reasoning tasks without the need for task-specific training (Zhang et al., 2024; Wang et al., 2024). These models, including CLIP (Radford et al., 2021), BLIP (Li et al., 2022), Flamingo (Alayrac et al., 2022), GPT-4V (Achiam et al., 2023), and Gemini 1.5 (Team et al., 2024), have achieved state-of-the-art performance in visual question answering, spatial localization, and cross-modal understanding. Growing emphasis on explainable multimodal reasoning has led to the development of models and interpretability techniques that enhance transparency in vision-language decision-making Tan (2025); Shen et al. (2025); Din et al. (2025). Unlike traditional vision-only deep learning models, VLMs integrate textual context directly into their predictions, enabling them to generate not only object detections but also interpretable, language-grounded explanations of visual content. These qualities have proven valuable in high-stakes domains such as medical imaging (Shi et al., 2025), remote sensing (Kuckreja et al., 2024; Weng et al., 2025), and robotics (Akiyama et al., 2024; Gao et al., 2024), where flexibility, domain adaptation, and explainability are crucial. Despite their considerable potential, VLMs have seen limited use in agricultural research, particularly in applications like weed detection and crop monitoring, where both spatial precision and contextual understanding are critical. Taking under consideration the capabilities of VLMs and the current limitations of UAV based weed detection workflows, these models offer a timely and largely unexplored opportunity to improve generalization, interpretability, and data-driven decision support in precision agriculture.

In this study, we explore the zero-shot capabilities of VLMs for weed detection and visual reasoning using real-world drone imagery collected from soybean fields. We systematically evaluate six leading commercial VLMs which are OpenAI’s GPT-4.1 and GPT-4o, Google’s Gemini Flash 2.5 and Flash Lite 2.5, and Meta’s LLaMA-4 based Scout and Maverick using a curated, ground-truth annotated dataset from prior agricultural field studies. Each model is prompted through a standardized zero-shot framework to perform multiple tasks, including weed presence detection, spatial localization, crop type classification, and crop growth stage estimation. To probe model robustness, we introduce a novel Error-Probing Prompting (EPP) strategy, which explicitly challenges models to revise their initial decisions under the assumption that weeds are present. Human experts evaluate model responses across five qualitative reasoning dimensions grounding, specificity, plausibility, non-hallucination, and actionability to assess interpretability and decision-making quality.

This paper advances the state of the art through the following contributions:

- Comprehensive evaluation of commercial Vision-Language Models for weed detection in drone captured agricultural imagery, under zero-shot conditions.
- Development of a standardized prompting framework that enables VLMs to produce interpretable, multi-task outputs encompassing weed detection, spatial localization, crop classification, and growth stage estimation.
- Introduction of an Error-Probing Prompting (EPP) strategy designed to evaluate a model’s ability to self-correct through feedback-driven reasoning, thereby simulating the kinds of interpretive errors encountered in real-world scenarios.
- Design and implementation of an expert-based evaluation protocol that assesses model interpretability across five practical criteria directly relevant to agricultural decision-making contexts.
- Comprehensive comparative analysis of six state-of-the-art VLMs, examining their performance trends, reasoning behaviors, and common failure cases, and providing insights into their readiness for deployment in autonomous crop management systems.

## 2 Related works

### 2.1 Weed detection in precision agriculture

Recent advancements in object detection have focused on improving robustness in visually complex environments, including overlapping targets and cluttered backgrounds Li et al. (2024a); Wang et al. (2025b); Xu et al. (2024). The integration of machine vision has become a cornerstone of modern, sustainable weed-management strategies that promote the use of diverse and complementary control methods (Qu and Su, 2024; Sarvakar and Thakkar, 2024). By combining advanced imaging sensors with computational algorithms, these systems can differentiate crops from weeds, identify specific weed species, detect plant anomalies or diseases, and support precision-based interventions for effective weed control. As the demand for accuracy and resource efficiency grows in agriculture, there has been increasing interest in robotic and vision-guided platforms capable of managing weeds throughout the active crop-growth stages (Gao et al., 2025; Suzer et al., 2024). Such approaches enable highly targeted treatments ranging from localized herbicide spraying to mechanical or thermal weed removal thereby minimizing chemical dependence and reducing the risk of herbicide-resistant weed populations (Bauer et al., 2020).

Traditional weed-control methods including manual weeding, mechanical tillage, biological control, and herbicide use remain widely used (Stanley and Entz, 2022; Jin et al., 2022; Gazoulis et al., 2021). Manual weeding, for instance, is labour intensive, time consuming, potentially loosens topsoil, and is ill suited for large scale modern agriculture. Mechanical weeding is faster and less labour intensive but may struggle to eradicate associated weeds comprehensively. Biological control holds ecological promise but remains slow in effect and at early stages of implementation. Herbicide use delivers broad scale efficacy and economic benefit, yet it carries significant environment and crop health trade offs pollution, herbicide resistant weeds, wasted resources, and crop yield impact (Rodrigo et al., 2014). These limitations reinforce the shift toward smarter, sensor driven vision based solutions.

In recent years, the field of weed detection has been revolutionised by deep-learning algorithms, particularly convolutional neural networks (CNNs). Models proposed in (Hasan et al., 2021; Ahmad et al., 2021; Villette et al., 2021; Hu et al., 2021) demonstrate strong performance in weed recognition tasks using CNNs. For example, study in (Asad and Bais, 2020) applied a maximum-likelihood classification step followed by CNN semantic segmentation (SegNet, U-Net) on high-resolution colour images of oilseed-rape fields, and reported an average intersection over union (IoU) of 0.8288 and a joint frequency-weighted IoU of 0.9869. Another study (Ramirez et al., 2020) used optical aerial imagery over sugar-beet fields in Germany and trained DeepLabV3 for weed segmentation; the model achieved AUC of 0.89 and F1 of 0.81. Further the study in (Saleem et al., 2022) uses an improved Faster R-CNN with custom anchor frame scaling and aspect ratios applied on apple weeds and achieved a 2.58% mAP increase, reaching an average precision of 24.95% for challenging weed classes.

Despite these advances, several challenges remain. High similarity between crops and weeds, differences between weed species, variable growth stages, sensor variations, and changing lighting or field conditions all reduce model performance Genze et al. (2023); Goel et al. (2024). Many approaches depend on large, carefully labeled datasets collected under controlled conditions, which makes it hard to scale or apply them to new crops and fields. Interpretability is also limited: models may provide detections but give little insight into how decisions are made. Machine-vision methods make up about 28% of weed-control solutions and deep learning about 19%, but gaps still remain in weakly-supervised learning, early detection of herbicide-resistant weeds, and real-world deployment of autonomous systems Goel et al. (2024).

Vision–language models (VLMs) have recently emerged as a complementary direction for agricultural perception tasks, offering stronger generalization, improved zero-shot results, and reduced dependence on large annotated datasets. Early work has shown that fine-tuned or adapted VLMs can achieve admirable zero-shot and few-shot weed-recognition performance (Yu et al., 2025). Other studies explore combining VLMs with federated learning to preserve data privacy across farms, demonstrating substantial gains in agricultural image understanding while reducing communication overhead (Li et al., 2025). Broader surveys of multimodal AI in smart agriculture further highlight VLMs as a promising future paradigm for tasks such as weed detection, crop-condition monitoring, and natural-language agronomic querying (Min et al., 2025). Meanwhile, new multimodal agricultural benchmarks such as AgMMU reveal that current VLMs still struggle with fine-grained perception and knowledge-intensive questions, such as distinguishing weeds within visually complex fields, thereby underscoring the need for improved reasoning and grounding capabilities (Gauba et al., 2025). These developments illustrate both the potential and the remaining challenges of applying VLMs to real-world open-field weed detection, motivating deeper investigation into prompting strategies, interpretability, and model adaptability.

These works present the challenges and highlight the need for weed-detection systems that are more adaptable, interpretable, and resilient systems that can operate with minimal human oversight while maintaining performance across diverse environments. Despite progress in deep learning–based weed detection, current systems still face major limitations in generalization, interpretability, and adaptability across varying field conditions. This demand creates an opportunity to explore advanced multimodal frameworks particularly vision–language models, which offer strong zero-shot capabilities, natural language explainability, and cross-domain generalization. VLMs present a promising direction toward more intelligent, scalable, and context-aware solutions for precision weed management.

### 2.2 UAVs and remote sensing for crop monitoring

Unmanned Aerial Vehicles (UAVs) have become an essential part of modern precision agriculture, offering high-resolution and flexible monitoring of crop growth, physiological health, and environmental conditions. Deep learning continues to advance agricultural scene understanding, with recent work demonstrating improved performance in crop-row segmentation and multi-sensor agricultural monitoring Chen et al. (2025); Nasir et al. (2025); Deng et al. (2025). With multispectral, hyperspectral, and thermal sensors onboard, UAVs can capture detailed measurements such as canopy temperature, vegetation indices, and spectral reflectance that reflect key physiological traits like chlorophyll levels, nitrogen content, and water-use efficiency (Sishodia et al., 2020; Yang et al., 2025; Ma et al., 2022). These capabilities have proven especially valuable for major crops like wheat, maize, rice, and soybean, where variations in soil fertility and moisture often result in uneven yields (Guebsi et al., 2024).

With these advanced sensing abilities, UAV-based remote sensing has become an important tool for spotting and classifying weeds in fields. Most research focuses on UAVs with RGB or multispectral sensors because they are small, easy to operate, and work well in different field conditions (Anderson and Gaston, 2013). Each type has its strengths RGB cameras are cheap and provide clear images but capture only limited spectral information, while multispectral sensors are more expensive but give richer data, helping to better distinguish weeds from crops (Maes and Steppe, 2019). Among the available sensor types, RGB cameras remain the most widely used in precision agriculture especially for weed identification because of their affordability, ease of integration, and strong compatibility with machine learning workflows for object based image analysis (Tsouros et al., 2019).

Huang et al. (Huang et al., 2018) demonstrated the potential of UAV-mounted RGB imagery for generating high-accuracy weed-cover maps to support site-specific weed management (SSWM) and optimize crop yield. Using a fully convolutional network (FCN), the study achieved an overall accuracy of 94% and a weed-recognition accuracy of 88%, illustrating the strength of deep learning models in extracting spatial and semantic features from aerial imagery. Likewise, Gašparović et al. (Gašparović et al., 2020) evaluated several manual and automatic classification methods using UAV-acquired RGB imagery and found that automatic classification achieved the highest overall accuracy of 89%. These results demonstrate the strong potential of RGB based UAV imagery, when combined with deep learning, to generate efficient and accurate weed maps for precision field management.

While RGB imaging remains predominant, there is a steady shift toward the adoption of multispectral systems for more advanced vegetation and weed analysis. Their superior spectral resolution supports the computation of a wider range of vegetation indices such as NDVI, SAVI, and GNDVI allowing more consistent temporal monitoring of crop development and stress progression (Esposito et al., 2021). This increased spectral sensitivity allows for the detection of subtle physiological changes linked to nutrient deficiencies, pest infestations, or disease. However, these benefits come at the expense of higher costs and more complex preprocessing and calibration steps—challenges that are generally not present with standard RGB imagery (Tsouros et al., 2019).

UAV based remote sensing has progressed from a traditional imaging tool to a key enabler of intelligent agricultural analytics. By integrating UAV acquired data with deep learning frameworks, researchers can now develop interpretable models that capture and analyze complex crop and weed dynamics. As research progresses toward multimodal and vision–language systems, UAV imagery serves as a critical visual input that can be semantically linked with textual agronomic knowledge to support context aware reasoning, explainable decision-making, and adaptive management in precision agriculture.

## 3 Methodology

**Figure 1:**
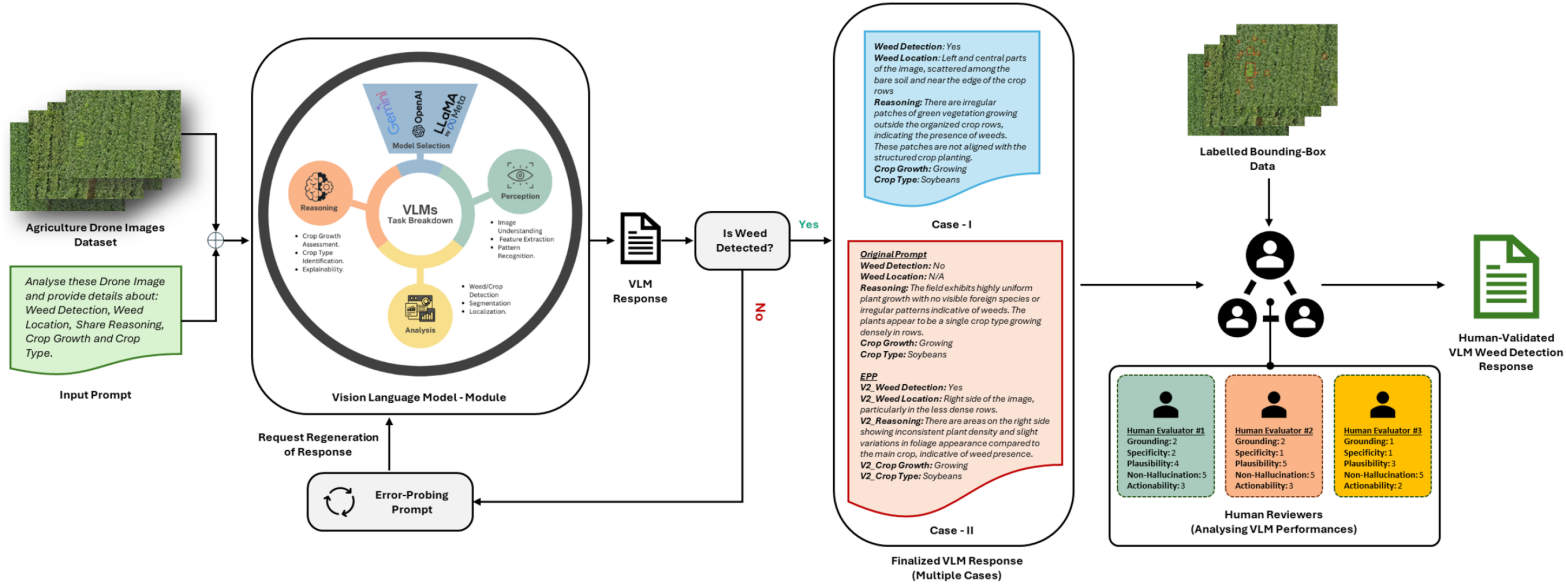
Overall architecture of the proposed vision-language model (VLM) evaluation framework for zero-shot weed detection in drone-based agricultural imagery. The process includes prompt formulation, model reasoning, error-probing, and human validation.

**Figure 2:**
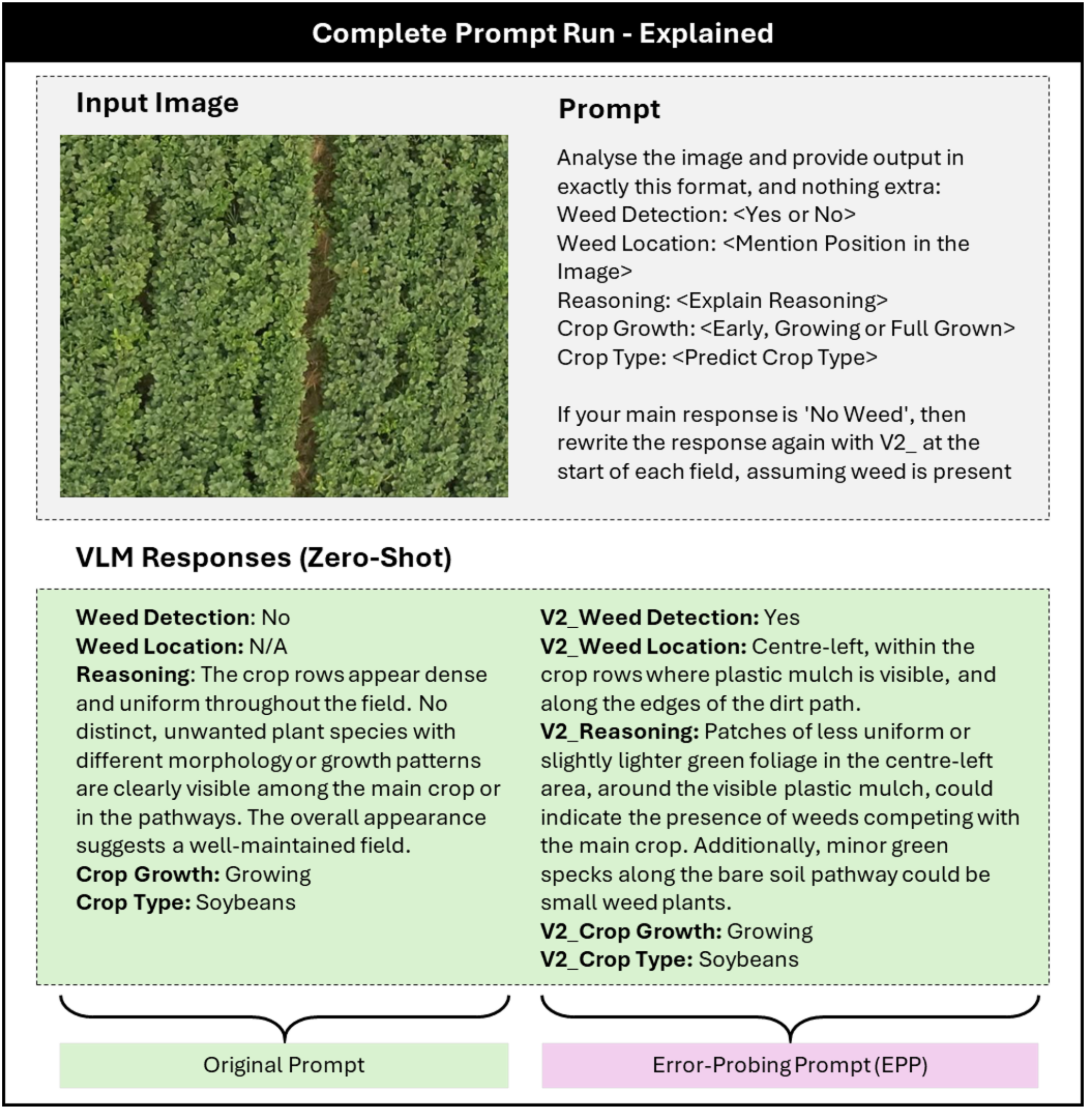
Detailed prompt interaction showing the original prompt (left) and error probing prompt (EPP) response (right). The model first provides an initial reasoning and is then compelled to reanalyze under the assumption that weeds are present.

This section details the proposed methodology designed to evaluate the zero-shot weed detection and reasoning capabilities of commercial Vision-Language Models (VLMs) using drone-based agricultural imagery. The workflow integrates image-based perception, structured prompting, error-probing strategies, and expert-based interpretability assessment. **Figure 1** illustrates the complete experimental pipeline, while **Figure 2** explains the detailed prompt interaction process between the image input and the VLM response generation.

### 3.1 Overview of the proposed framework

The proposed methodological framework is organized into five main components, (1) dataset curation and ground-truth alignment, (2) standardized zero-shot prompting, (3) VLM inference with integrated error probing, (4) human-centered interpretability assessment, and (5) comparative performance evaluation. These modules operate within a closed-loop evaluation cycle that facilitates both quantitative and qualitative analyses of each VLM’s capability to detect weeds, identify crop types, and produce contextually coherent reasoning.

### 3.2 Dataset description and ground truth alignment

The dataset employed in this study builds upon the work of (Rehman et al., 2024), comprising a curated set of 441 drone-acquired RGB images collected from soybean fields. It was specifically designed to facilitate agricultural scene understanding tasks, including weed localization and crop growth assessment. Each image in the dataset is accompanied by manually annotated bounding boxes that precisely delineate the weed regions.

Let the dataset be denoted as $D={\left\{(I_{i},B_{i})\right\}}_{i=1}^{N}$, where *I_i_* is the *i*-th drone image and $B_{i}=\{b_{i1},b_{i2},\ldots ,b_{in_{i}}\}$ represents the set of ground-truth weed bounding boxes. Each bounding box *b_ij_* is parameterized as:

(1) $$b_{ij}=(x_{min},y_{min},x_{max},y_{max})$$

where (*x_min_,y_min_*) and (*x_max_,y_max_*) correspond to the top-left and bottom-right coordinates of the rectangular region, respectively. To ensure consistency across models, all images were resized to 640 × 640 pixels. No additional data augmentation was performed to maintain the zero-shot integrity of the evaluation.

### 3.3 Vision-language model selection and configuration

Six commercially available VLMs were selected for evaluation, Gemini Flash 2.5 and Gemini Flash Lite 2.5 (Google) (Team et al., 2024), LLaMA 4 Scout and LLaMA 4 Maverick (Meta) (Dubey et al., 2024), and ChatGPT 4.1 (Achiam et al., 2023) and ChatGPT 4o (Hurst et al., 2024) (OpenAI). Together, these models represent the current spectrum of multimodal perception and reasoning frameworks, spanning high-throughput fused token architectures, adapter-based vision-to-language alignment, and unified end-to-end embedding strategies. Their inclusion enables a broad comparative analysis of how architectural design choices influence zero-shot performance on agricultural imagery.

The Gemini Flash series focuses on efficient joint visual–text encoding. Gemini Flash 2.5 employs adaptive token compression and local patch aggregation to support dense scene understanding while maintaining low inference latency. Its lighter version, Gemini Flash Lite 2.5, keeps the same multimodal backbone but uses stronger pruning and quantization to reduce memory use and improve speed for devices with limited resources. In both models, visual regions are converted into compact token representations and processed together with text tokens, generating structured text outputs that help with interpretability.

LLaMA 4 Scout and LLaMA 4 Maverick extend Meta’s LLaMA framework to multimodal contexts through visual adapter modules that project image features into the language token space. The Scout configuration applies a streamlined projection pipeline to achieve stable cross-attention between modalities with minimum disruption to the base model. Maverick further integrates context conditioned visual fusion layers that adapt attention based on prompt semantics, allowing more selective focus on agricultural indicators such as irregular vegetation patterns or pathway artifacts suggestive of weed presence.

The ChatGPT 4.1 and ChatGPT 4o models reflect OpenAI’s recent advances in multimodal reasoning. ChatGPT 4.1 is built for high-accuracy reasoning and reliable instruction following, using a contrastively aligned image encoder with a strong autoregressive decoder. In contrast, ChatGPT 4o is designed for faster, more flexible interactions, with improved bidirectional image–text attention. This setup works especially well for counterfactual and corrective reasoning, making it a good fit for the Error Probing Prompting (EPP) framework used in this study.

For each model ℳ*_k_* in the evaluation set, a single standardized zero-shot prompt *P* (see Section 3.4) was applied to every image *I_i_*, generating a structured textual response *R_i_* as follows:

(2) $$R_{i}=M_{k}(I_{i},P).$$

Each model response *R_i_* was parsed into five subfields following the established evaluation protocol: weed detection (binary classification), weed localization (text-based spatial reference), reasoning justification (free-text explanation), crop growth stage (categorical), and crop type identification (categorical). To preserve strict zero-shot conditions, none of the models underwent task specific fine-tuning, received exemplar based prompting, or utilized any image level pre-processing beyond the standard resizing procedure. This consistent, untuned experimental setup enables a fair assessment of the inherent multimodal reasoning and visual grounding capabilities of all six commercial VLMs.

### 3.4 Zero-shot prompting framework

To ensure a fair and consistent evaluation of all VLMs, the same fixed zero-shot prompt was used for every image–model pair. This helps reduce variability from different prompt designs and gives a clearer view of each model’s visual reasoning and language understanding. The standardized prompt asked each model to provide five specific outputs, including weed detection, weed localization, reasoning explanation, crop growth stage, and crop type, all within a predefined response format.

The standardized prompt used was as follows:

*“Analyze the image and provide output strictly in this format and nothing extra: Weed Detection: <Yes or No> Weed Location:<mention Position in the Image> Reasoning: <Explain Reasoning> Crop Growth: <Early, Growing, or Fully Grown> Crop Type: <Predict Crop Type>“*

This fixed structure ensured consistent interpretability across VLMs while minimizing linguistic bias. Each model ℳ*_k_* received the same input pair $\left(I_{i},P\right)$, where $I_{i}$ is the *i^th^* image and *P* is the fixed prompt, yielding a structured textual output $R_{i}=M_{k}(I_{i},P)$. Using a single prompt ensured both syntactic and semantic consistency across all outputs, making it easier to directly compare the models.

By applying a unified zero-shot prompting framework, the evaluation focused on each model’s inherent ability to detect weeds, perform visual reasoning, and interpret agricultural scenes without relying on task specific fine-tuning or adaptive prompt adjustments. This consistent setup provided a controlled baseline for the subsequent implementation of the Error Probing Prompting (EPP) mechanism.

### 3.5 Error probing prompting mechanism

While zero-shot evaluation shows the natural reasoning abilities of VLMs, it does not test their capacity to self-correct or adapt when given feedback. To address this, we introduce the Error Probing Prompting (EPP) mechanism, an auxiliary prompting strategy that evaluates a model’s self-correction, reasoning consistency, and context-sensitive adaptability.

Prior works has explored several families of prompting strategies to improve reasoning robustness, including chain-of-thought with self-consistency (Wang et al., 2022), Tree-of-Thoughts search over alternative reasoning branches (Yao et al., 2023), reflective agents that incorporate self-critique (Shinn et al., 2023), and counterfactual prompting for robustness analysis (Li et al., 2024b). In agriculture, prompting has been used to guide LLMs and VLMs for crop disease diagnosis and domain adaptation, for example through Chain-of-Thought prompting in (Pan et al., 2025) or expert-tuned instruction datasets in (Awais et al., 2025).

Building on these ideas, Error-Probing Prompting (EPP) can be viewed as a domain-specific counterfactual probe. In the EPP framework, each model first analyzes an image *I_i_* using the standard fixed prompt *P*, producing an initial response $R_{i}^{\left(1\right)}$. If the model reports “No Weed Detected,” a second probe prompt $P_{\text{EPP}}$ is issued, asking the model to re-examine the same image under the assumption that weeds are present. This conditional re-evaluation mimics a feedback-driven reasoning process, similar to real-world scenarios where models may receive corrective input from human operators or automated detection systems. The two-stage prompting sequence can be formalized as follows.

(3) $$R_{i}^{\left(1\right)}=M(I_{i},P),$$

(4) $$R_{i}^{\left(2\right)}=M(I_{i},P_{\text{EPP}}),$$

where M denotes the VLM, P is the fixed baseline prompt, and $P_{\text{EPP}}$ is the modified error probing version.

The $P_{\text{EPP}}$ prompt retains the original structured response format but adds a counterfactual reasoning clause, such as:

*“Assume that the image contains weeds, even if they are not immediately visible. Re-analyze the image carefully and provide your reasoning based on this assumption.”*

This carefully designed adjustment prompts the model to re-examine its visual reasoning, helping to identify weed regions that might have been missed or interpreted ambiguously. The EPP mechanism functions as a stress test for model adaptability, evaluating whether a VLM can move beyond its initial, rigid decision boundaries and engage in higher-order reasoning.

### 3.6 Human expert evaluation protocol

To systematically evaluate the clarity and practical relevance of model explanations, we define five qualitative reasoning dimensions that capture different aspects of visual reasoning quality. These dimensions are grounding, specificity, plausibility, non-hallucination, and actionability. These dimensions operationalize different aspects of multimodal reasoning quality in agricultural settings. The detailed scoring rubric is provided below.

- Grounding evaluates whether a model’s explanation is anchored to concrete, visible evidence in the image. High scores (5) indicate that the model explicitly references identifiable visual features such as color, shape, or spatial position linked to verifiable regions within the frame. Low scores (1) are assigned when evidence is absent, vague, or not visually traceable.
- Specificity measures the precision of spatial and descriptive references. Responses that identify locations using quantifiable or structured indicators (e.g., “row 2, center-left, approximately 0.3 of the frame width”) receive high scores, while those relying on imprecise language (e.g., “somewhere on the left”) score lower. This dimension captures how effectively a model communicates localizable and reproducible cues.
- Plausibility captures whether the reasoning expressed by the model aligns with agronomic logic and observable field conditions. A plausible explanation correctly relates visual cues to biological or environmental phenomena for instance, recognizing that lighter leaf color near field edges suggests herbicide drift rather than disease. Scores of 5 denote consistent, domain-appropriate reasoning; scores of 1 denote implausible or contradictory logic.
- Non-Hallucination assesses factual fidelity between textual claims and visual evidence. Models that refrain from describing unobservable, imagined, or misidentified elements receive higher scores. This criterion is especially critical for safety-sensitive agricultural applications, where fabricated objects (e.g., “weeds” not actually visible) can mislead decision-making.
- Actionability evaluates whether the model’s explanation offers practical guidance for field verification or intervention. A response scoring 5 provides concrete next steps or operational cues (e.g., “inspect the second row from the left for broadleaf weeds with purple stems”), while a score of 1 indicates generic or non-instructive statements. This dimension emphasizes the applied utility of AI reasoning for human decision support in precision agriculture.

Each response pair $\left(R_{i}^{\left(1\right)},R_{i}^{\left(2\right)}\right)$ was independently evaluated by three domain experts across these five dimensions. Experts were comprised of two Agronomists with experience in soybean and weed-management research, and one experienced Farmer with over a decade of operational field management experience. All experts were trained on the rubric, provided example ratings, and instructed to evaluate model responses without knowledge of which VLM generated them. Each metric was rated on a 1–5 Likert scale, where higher scores indicate greater interpretability. These metrics were used to verify that expert ratings were sufficiently consistent to justify aggregating them. The merged score therefore reflects a stable representation of expert agreement rather than individual annotator variability.

The final evaluation score for a given model *M* was computed as the mean of normalized scores across all experts and test samples:

(5) $$E(M)=\frac{1}{N\times K}\sum_{i=1}^{N}\sum_{k=1}^{K}\frac{G_{ik}+S_{ik}+P_{ik}+H_{ik}+A_{ik}}{5}$$

where *N* is the number of test samples and *K* = 3 is the number of evaluators. Inter-rater consistency was measured using Cohen’s Kappa (*κ*), to quantify pairwise agreement on categorical scoring tendencies, and Cronbach’s Alpha (*α*) to measure internal consistency across the five scoring dimensions and to validate scoring reliability.

### 3.7 Quantitative weed localization evaluation

For cases where the VLM provides spatial localization in textual form (e.g., “top-left”, “center-row”), we evaluate these qualitative descriptions by having human annotators interpret them as regions of interest on the image. This human-interpreted ROI is then compared with the ground-truth weed bounding boxes using a region-overlap approximation, allowing us to visualize the region-overlap approximation. For the text-to-region matching score *S_m_* is given by:

(6) $$S_{m}=\frac{A_{\text{overlap}}}{A_{\text{union}}}$$

where *A*_overlap_ is the intersection area between the described region and the ground-truth weed region, and *A*_union_ represents their combined area. This metric provides an interpretable IoU-like measure for text-based spatial reasoning. However in practice, VLM outputs contained substantial variability and ambiguity, especially in zero-shot settings and during EPP. As a result, *S_m_* values were unstable and not sufficiently reliable for formal comparison across models. We therefore used *S_m_* internally as a diagnostic to confirm that interpretability patterns aligned with spatial grounding, but we did not present it as a primary metric. Our emphasis instead remains on actionable, human-interpretable regional localization.

## 4 Results and discussion

### 4.1 Zero-shot evaluation of VLMs in agricultural tasks

We conducted a zero-shot evaluation of six commercial VLMs across several key agricultural tasks which are weed detection, crop growth stage estimation, crop type classification, and weed localization with reasoning. In addition, we introduced an Error Probing Prompting (EPP) strategy to assess each model’s ability to self-correct. **Table 1** summarizes the performance of each model across multiple metrics, including qualitative reasoning scores rated by human evaluators.

**Table 1:** Performance of vision-language models (VLMs) under zero-shot and error-probing prompting (EPP) settings.

Model	WD acc	CG acc	CT acc	Zero-shot: weed localization accuracy + VLM reasoning (0–5)					Self-corr.	EPP: weed localization accuracy + VLM reasoning (0–5)				
				Grounding	Specificity	Plausibility	Non-Hall.	Actionability		Grounding	Specificity	Plausibility	Non-Hall.	Actionability
ChatGPT-4.1	0.3083	0.1927	0.8322	2.0294	1.9191	4.6985	4.9264	2.4411	1.00	1.0263	1.0098	3.7540	4.9409	1.6852
ChatGPT-4o	0.4489	0.7857	0.7103	1.3737	1.2575	4.1767	4.9494	1.9141	0.9917	0.6413	0.6118	3.2827	4.7468	1.3417
LLaMA-4 Scout	0.1496	0.0023	0.3946	1.4545	1.3333	4.1818	4.8787	1.9545	0.6773	0.7500	0.7830	2.9952	4.4811	1.2028
LLaMA-4 Maverick	0.4557	0.8705	0.5306	1.5074	1.4378	4.2935	4.8557	2.0845	1.0000	0.4333	0.4416	3.1083	4.4916	1.1833
Gemini Flash 2.5	0.7460	0.0493	0.8753	2.2431	2.0668	4.5379	4.8936	2.6200	0.7857	1.3977	1.4090	3.8068	4.5454	1.8863
Gemini Flash Lite 2.5	0.6825	0.0000	0.9048	1.7076	1.5581	4.3820	4.7408	2.1794	0.1142	0.0000	0.0000	0.0000	0.0000	0.0000

WD, Weed Detection; CG, Crop Growth; CT, Crop Type; EPP, Error-Probing Prompting. Scores are averaged across annotators. “Self-Corr.” denotes how often the EPP prompt changed the VLM response from ‘No’ to ‘Yes’. Reasoning scores are rated from 0 (poor) to 5 (excellent) by expert annotators.

Overall, Gemini Flash 2.5 exhibited the strongest zero-shot performance in both classification and reasoning metrics, particularly excelling in interpretability (actionability and plausibility). ChatGPT-4.1 showed high reasoning scores but struggled with basic detection accuracy. ChatGPT-4o balanced moderate detection capabilities with competitive reasoning, while LLaMA-4 Maverick performed better than Scout in classification but underperformed in localization.

A notable observation is the absence of usable EPP reasoning data for Gemini Flash Lite 2.5. While this model achieved high weed detection accuracy, its EPP responses were non-informative. Specifically, it produced 16 “Yes” responses in EPP that were exact repetitions of its original “Yes” predictions. These were classified as prompting errors and not usable for evaluating reasoning behavior. As such, all EPP reasoning scores for Gemini Flash Lite 2.5 were set to 0, and its reasoning was evaluated only under standard zero-shot prompts.

These results illustrate the variability across models not just in prediction accuracy but also in their ability to provide contextual, grounded, and actionable explanations a crucial feature for practical adoption in precision agriculture systems.

### 4.2 Error and prompting behavior analysis

Although quantitative accuracy and reasoning scores offer a broad indication of overall VLM performance, analyzing failure modes is crucial for uncovering underlying limitations and informing future enhancements. To explore this, we conducted a detailed analysis of the error patterns observed under both standard zero-shot prompting and the EPP framework. We observed that a single response can exhibit multiple error types (i.e., both mis-localization and incorrect reasoning); therefore, we treat each image response pair as a multi-label instance, allowing it to contribute to multiple error categories. **Table 2** summarizes the primary categories of errors, along with their frequencies and proportional distributions across all evaluated models.

**Table 2:** Prompting and reasoning errors observed during VLM evaluation.

Error type	Gemini Flash	Flash Lite	LLaMA Maverick	LLaMA Scout	ChatGPT-4o	ChatGPT-4.1
Repeating Response in EPP	–	17 (3.85%)	–	40 (9.07%)	4 (0.9%)	–
Unable to Generate EPP	23 (5.2%)	138 (31.29%)	–	168 (38.09%)	–	–
Only Gives EPP, Not Original	15 (3.4%)	–	–	–	–	–
Minor Prompt Errors	–	4 (0.9%)	202 (45.8%)	–	–	–
Unable to Analyze Image	–	–	–	–	9 (2.04%)	–
Total Errors	38 (8.61%)	159 (36.05%)	202 (45.8%)	208 (47.16%)	13 (2.94%)	–

Percentages denote the share of total evaluations for each model.

Some models, particularly Gemini Flash Lite and LLaMA Scout, often repeated their original responses during EPP, even when a correction was expected. This repetition limits the effectiveness of EPP because it prevents the model from exploring uncertainty or adjusting its outputs based on the new prompt. The most common issue across several models was a complete failure to produce a distinct response during the EPP phase. This was especially noticeable in Gemini Flash Lite (31.29%) and LLaMA Scout (38.09%), indicating potential weaknesses in prompt chaining, response retention, or multi-turn visual reasoning. In some cases, such as with Gemini Flash, the model generated an EPP response but did not reproduce the corresponding output from the original prompt. While this did not always invalidate the results, it occasionally disrupted the evaluation process and made consistency checks and comparisons across models more difficult.

LLaMA Maverick frequently showed minor misinterpretations and inconsistencies in its prompts, affecting nearly half of its responses. These issues mainly involved partial template substitutions, omission of contextual cues, or difficulties distinguishing between crop and weed references. In contrast, ChatGPT-4o rarely but critically failed to analyze or interpret certain images, producing fallback or overly generic descriptions instead of engaging with the visual content. This points to possible issues in image preprocessing or internal vision–language integration.

Gemini Flash Lite 2.5 deserves special mention. While it performed well on standard detection metrics, its reasoning under the EPP framework was essentially absent. All sixteen affirmative (“Yes”) responses recorded during EPP were direct repetitions of the initial prompt, resulting in a complete reasoning penalty. As a result, all EPP reasoning scores for this model were set to zero, and only outputs from the original prompt were used for interpretability evaluation.

### 4.3 Comprehensive VLM performance on single-image analysis

**Figure 3:**
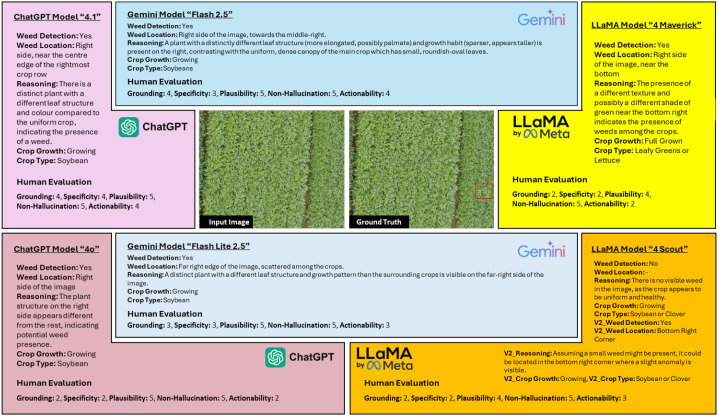
Comprehensive analysis of VLM predictions and human evaluation for a single test image, comparing models on reasoning quality, weed localization, and interpretability.

**Figure 3** presents a detailed comparison of six Vision-Language Models (VLMs) applied to a representative soybean field image. Each model’s zero-shot prediction includes textual reasoning, weed detection outputs, and human evaluation scores across five interpretability metrics.

Gemini Flash 2.5 showed the highest grounding and plausibility, scoring 5 out of 5, and accurately identified weed regions matching the ground-truth annotations. ChatGPT-4.1 also performed well in interpretability, providing a good balance of precise weed localization and clear reasoning. In contrast, LLaMA-4 based models exhibited weaker localization performance and lower specificity. LLaMA-4-Maverick occasionally confused canopy texture variations with weeds, while LLaMA-4 Scout often failed to detect any anomalies, returning “No Weed” in uniform fields. **Figure 3** underscores how interpretability correlates with visual-text alignment quality: models capable of grounding linguistic tokens in pixel regions (Like: Gemini Flash 2.5 and ChatGPT-4.1) generate more contextually valid reasoning.

### 4.4 Impact of error probing prompting on model sensitivity

To assess the model’s ability to reason and self-correct, the Error Probing Prompting (EPP) mechanism was applied after the initial inference stage. EPP focuses on cases that were previously classified as negative or uncertain, prompting the model to re-examine each image under the counterfactual assumption that weeds are present. This iterative feedback allows the model to revisit its earlier reasoning and potentially recover missed detections.

**Figure 4:**
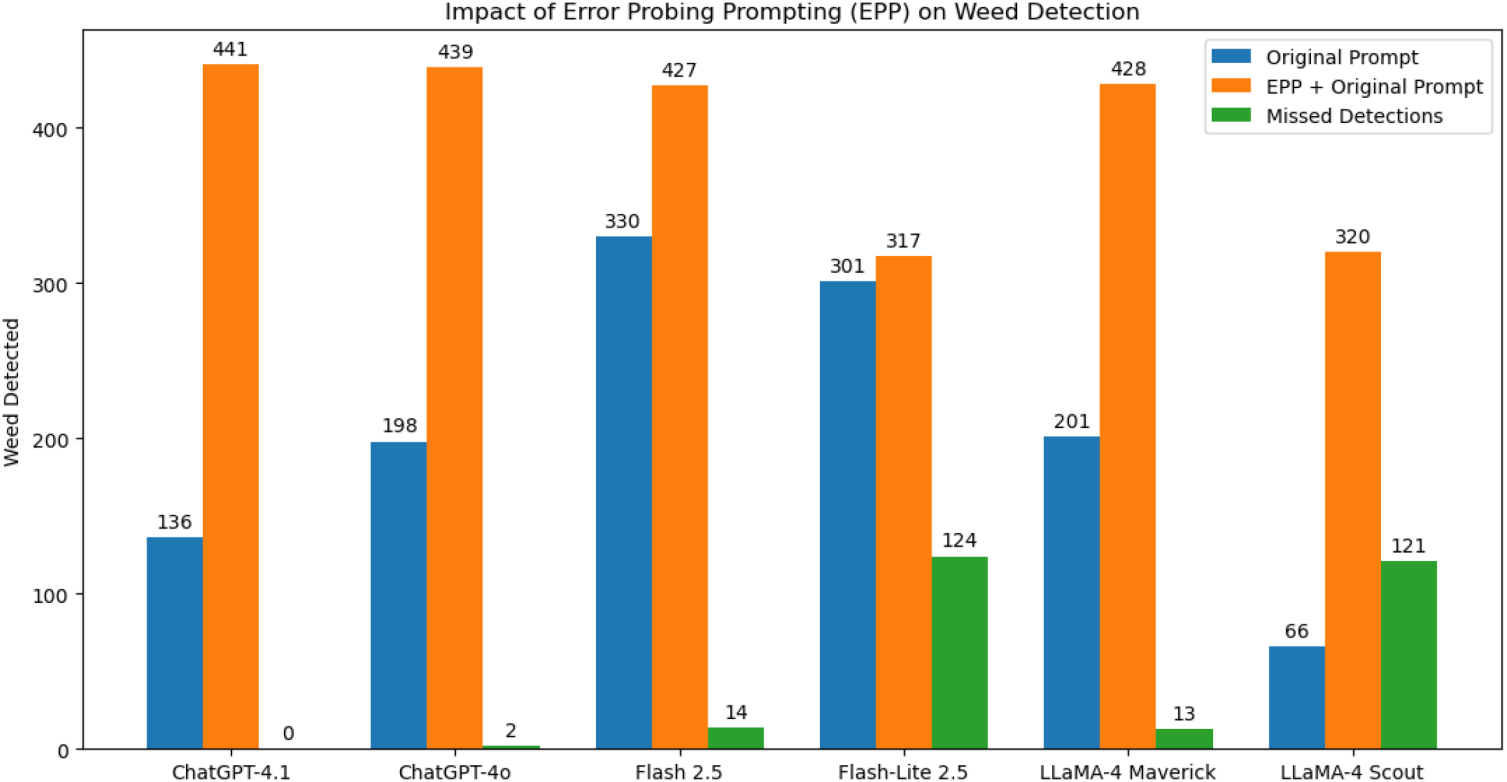
Comparison of weed detections using the original zero-shot prompt versus the Error Probing Prompting (EPP) approach. EPP prompts the model to re-evaluate images under the assumption that weeds are present, enabling recovery of previously missed detections.

**Figure 4** compares weed detections from the original prompt with those under the EPP condition. Across models, EPP consistently increased detection counts by addressing false negatives rather than simply inflating overall sensitivity. ChatGPT-4.1 showed the greatest improvement, detecting 305 previously missed instances, demonstrating strong adaptability to guided feedback. ChatGPT-4o also improved noticeably, reflecting balanced responsiveness without overcorrection.

Gemini Flash 2.5 maintained a high baseline performance and showed moderate gains under EPP, suggesting its precision oriented reasoning leaves fewer errors to correct. Gemini Flash Lite 2.5 saw minimal improvement, consistent with its lightweight design and limited capacity for iterative reasoning. Among the LLaMA-4 variants, Maverick recovered 227 new cases, while Scout recovered 254, indicating that although EPP enhances recall, the extent of improvement depends on each model’s reasoning depth and ability to integrate feedback effectively.

### 4.5 Visual grounding and human evaluation of model outputs

**Figure 5:**
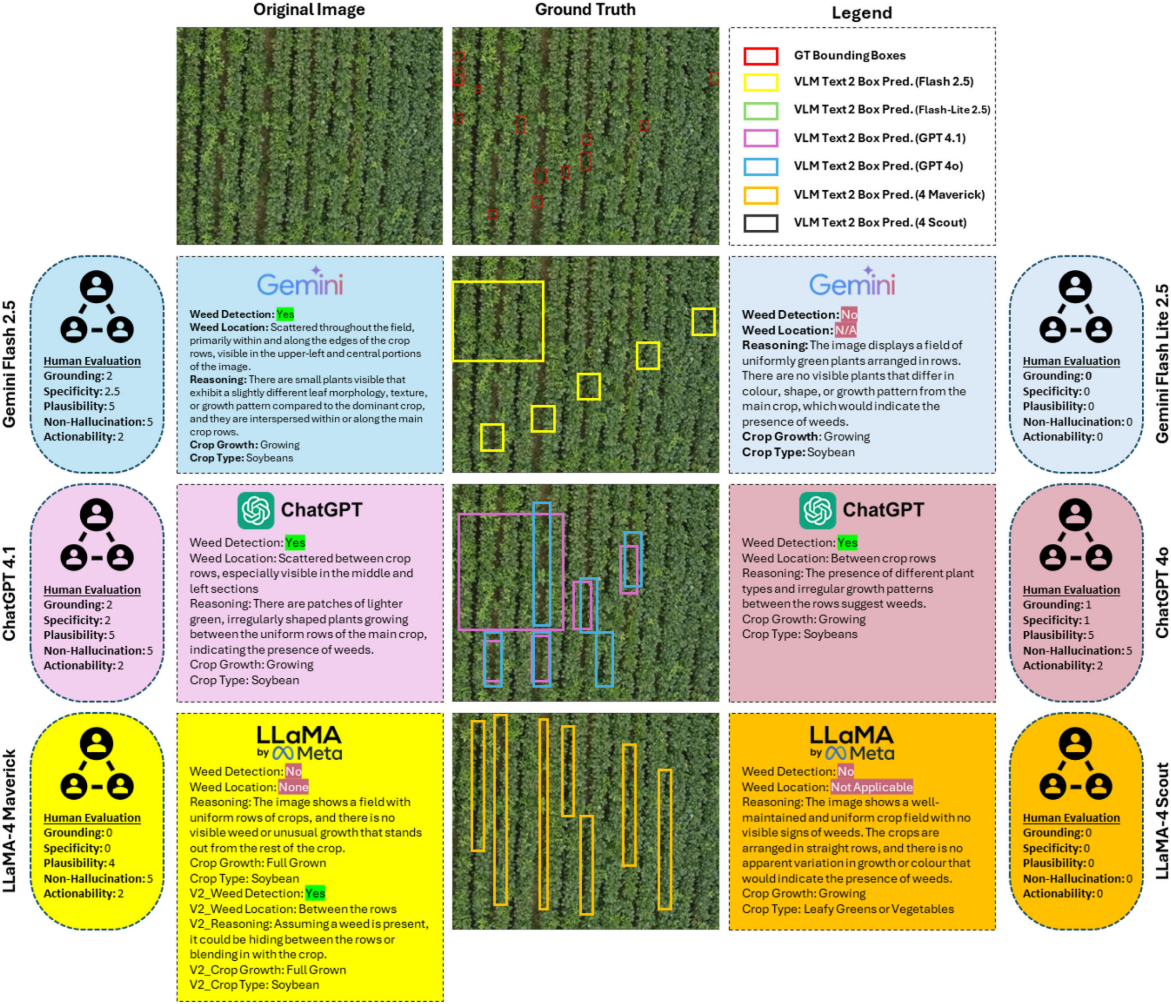
Visualization of VLM predictions overlaid on the ground-truth images. Color-coded bounding boxes distinguish model-predicted regions from annotated weeds, while human evaluation scores reflect interpretability based on defined criteria.

**Figure 5** visualizes how each model’s textual predictions correspond to spatial ground truth weed annotations. Ground-truth bounding boxes (in red) are overlaid alongside VLM predicted weed regions inferred from text descriptions.

Gemini Flash 2.5 and ChatGPT-4.1 achieved the closest alignment between described locations and annotated weed regions, demonstrating high grounding and plausibility. Conversely, LLaMA-4 Scout failed to detect weeds even when visible, while Gemini Flash Lite 2.5 produced empty or ambiguous responses. These results confirm that interpretability metrics especially Grounding and NonHallucination align with visual overlap accuracy, validating the human evaluation framework.

### 4.6 VLM parameter, performance, and compute comparison

**Figure 6:**
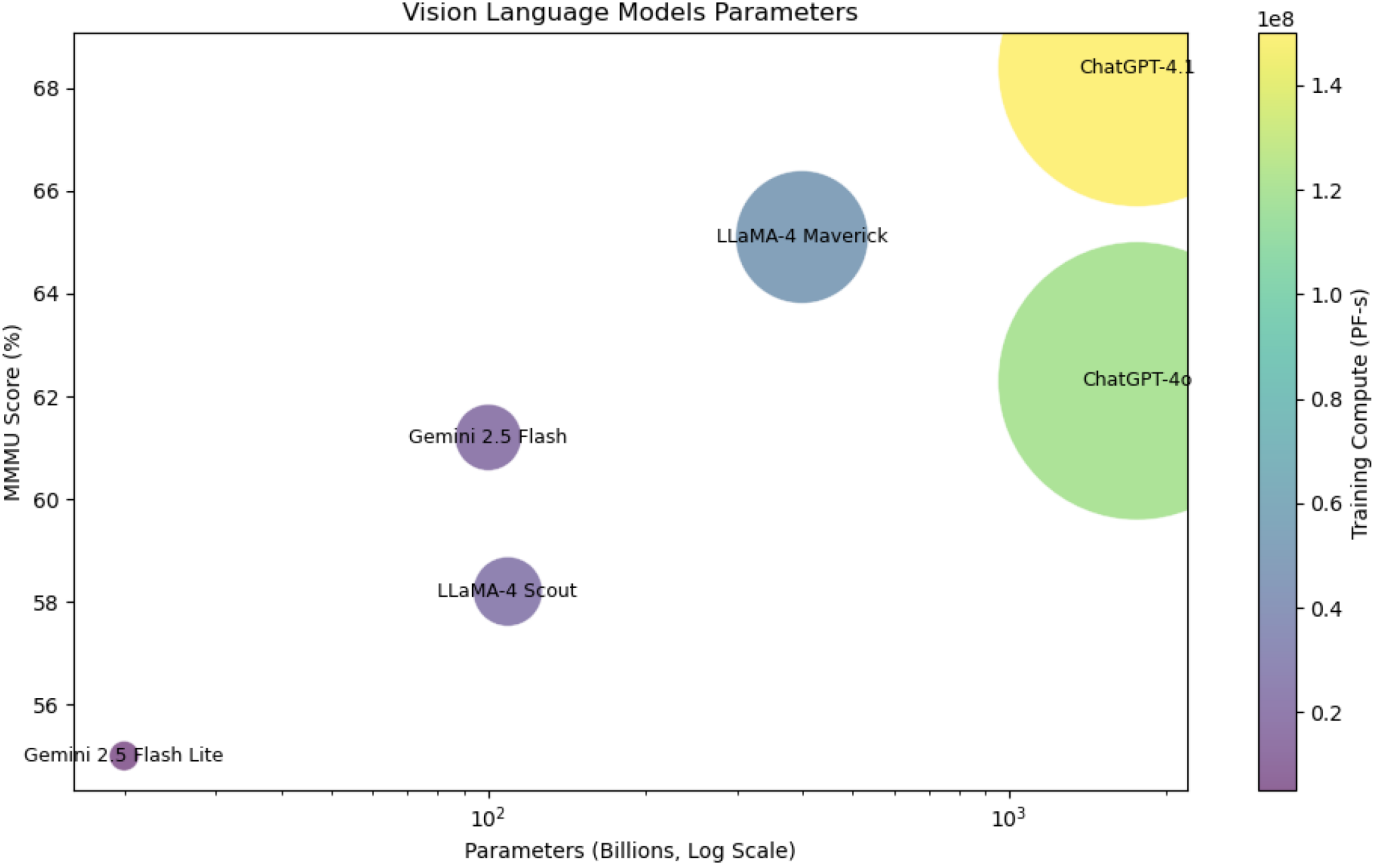
Comparison of VLM parameters, training compute, and general performance (MMMU score). Bubble size corresponds to compute scale, illustrating efficiency–performance trade-offs.

To connect model performance in agricultural tasks with overall scale, **Figure 6** shows the relationship between parameter count, training compute, and general reasoning ability, measured by the MMMU (Massive Multi-discipline Multimodal Understanding) benchmark (Yue et al., 2024). It is a large-scale evaluation score designed to measure a model’s general reasoning ability across diverse domains. It contains expert-level problems spanning science, engineering, humanities, and visual reasoning tasks, requiring models to integrate both textual and visual information. Higher MMMU scores indicate stronger cross-domain reasoning capabilities beyond narrow, task-specific performance. The bubble chart indicates that larger models, such as ChatGPT-4.1 and ChatGPT-4o, consistently achieve higher interpretability and more stable reasoning. Interestingly, Gemini Flash 2.5, though relatively small, provides strong interpretability, showing that good architectural design and effective cross-modal token alignment can matter more than model size. In contrast, smaller, heavily pruned versions like Gemini Flash Lite 2.5 show a noticeable drop in reasoning ability, highlighting the trade-off between computational efficiency and contextual understanding.

### 4.7 Expanded multi-image comparative analysis

**Figure 7:**
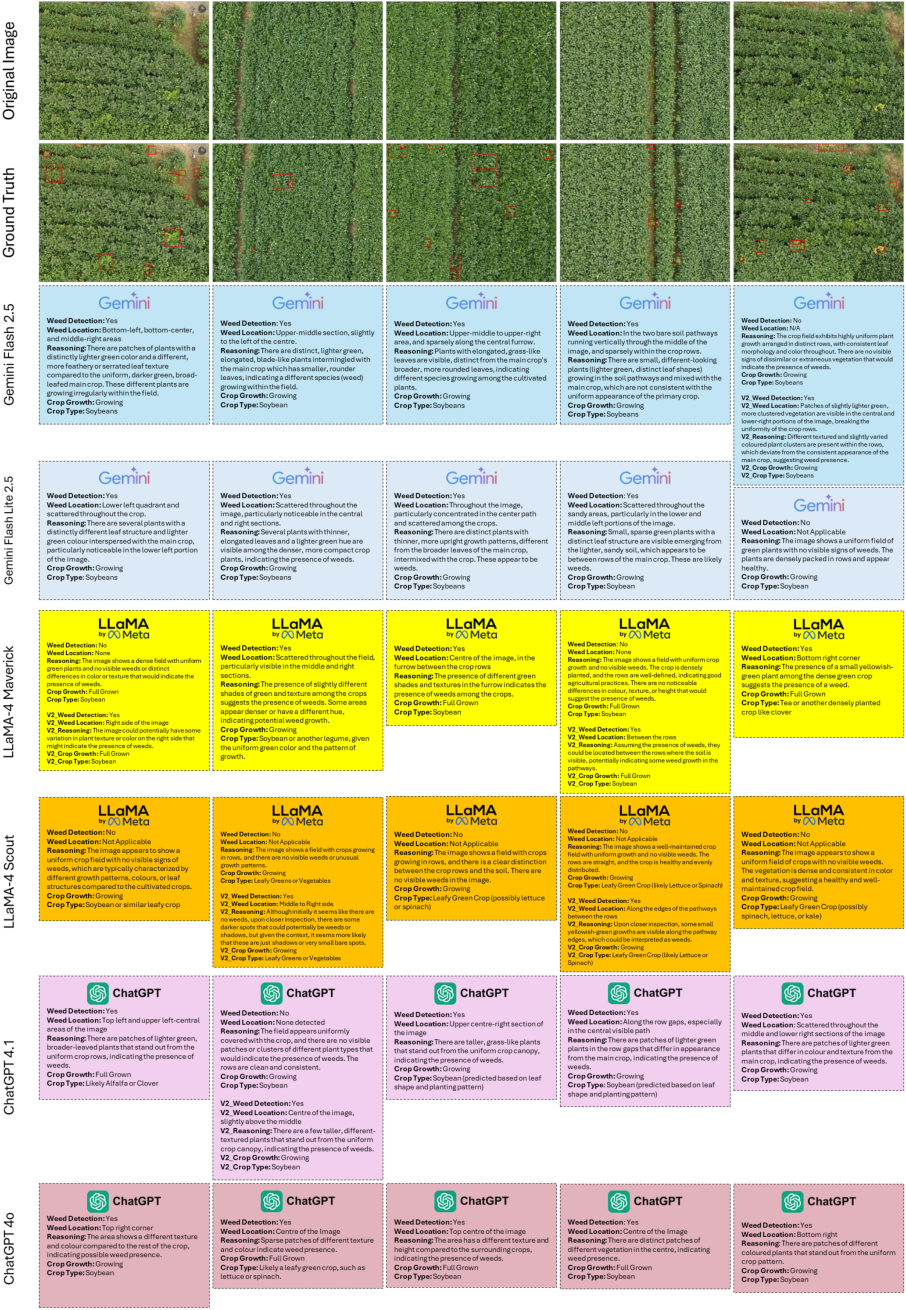
Expanded comparison of VLM outputs across multiple test images, showing both zero-shot and EPP responses. The results highlight each model’s consistency, interpretability, and range of reasoning capabilities.

**Figure 7** shows a detailed comparative analysis across multiple field images, highlighting each model’s predictions under both the original and EPP conditions. Gemini Flash 2.5 consistently delivered accurate weed detections and precise text-based localization across a variety of conditions, while the LLaMA-4 models showed considerable variability between test cases. Both ChatGPT-4.1 and ChatGPT-4o exhibited strong reasoning abilities, though they occasionally misinterpreted sparse canopy gaps as weed regions. These findings indicate that multimodal interpretability, rather than accuracy alone, provides the most reliable measure of real-world performance and robustness in agricultural applications.

### 4.8 Discussion and future works

This study highlights the potential of commercial VLMs for zero-shot weed detection and interpretability, yet several limitations remain. Our findings rely on a single soybean dataset captured with RGB UAV imagery, and performance may differ substantially across other crops, weed species, sensor types, and environmental conditions; modalities such as multispectral or hyperspectral imaging may reveal additional strengths or weaknesses in VLM reasoning. Because this work exclusively evaluates closed-source commercial models, the behavior of open-source VLMs and the influence of architectural factors such as vision encoders, alignment mechanisms, and training data, remains unexplored. Although some VLMs can output coordinates, their inconsistent and unreliable zero-shot spatial localization led us to adopt qualitative region-based evaluation, leaving opportunities for future prompting strategies and multimodal input–output formats. Rapid progress in AI has also introduced more capable models (e.g., Gemini 2.5 Pro, Grok-4, Anthropic’s Claude models), which we excluded due to cost and throughput constraints required for consistent, full dataset evaluation. Future research should expand benchmarks to diverse environments and sensor modalities, incorporate transparent open-source VLMs for architectural analysis, evaluate higher-capacity models as resources permit, refine spatial-localization and adaptive prompting techniques (including multi-turn EPP), and explore human-in-the-loop workflows to translate VLM reasoning into practical, scalable precision-weed-management systems.

## 5 Conclusion

This study demonstrates that modern vision–language models (VLMs) hold substantial promise for advancing automated weed detection and visual reasoning in precision agriculture. By leveraging their multimodal understanding, these models can interpret complex UAV imagery without task-specific training providing both analytical flexibility and human interpretable explanations. Through a unified zero-shot prompting framework and the introduction of Error Probing Prompting (EPP), we systematically evaluated six leading commercial VLMs, revealing key differences in accuracy, interpretability, and adaptability. Our findings show that Gemini Flash 2.5 achieved the best overall balance between detection accuracy and interpretive quality, while ChatGPT-4.1 and ChatGPT-4o excelled in reasoning depth and responsiveness to corrective prompts. Conversely, LLaMA-4 variants exhibited inconsistent spatial grounding, and Gemini Flash Lite 2.5, though computationally efficient, struggled with self-correction under feedback. These results suggest that model architecture and reasoning adaptability outweigh raw parameter count in determining agricultural reliability.

Beyond benchmarking, this work highlights interpretability as a key determinant of trust and usability in AI-assisted agronomy. The EPP mechanism proved especially valuable for exposing latent reasoning flexibility an essential trait for real-world deployment where environmental uncertainty and human-in-the loop decision-making prevail.

## Data Availability

The original contributions presented in the study are included in the article/supplementary material. Further inquiries can be directed to the corresponding authors. The data can be accessed from following repository: https://github.com/m-fahad-nasir/VLM_Weed_Framework.

## References

[B1] Achiam J. Adler S. Agarwal S. Ahmad L. Akkaya I. Aleman F. L. . (2023). Gpt-4 technical report. arXiv.

[B2] Ahmad A. Saraswat D. Aggarwal V. Etienne A. Hancock B. (2021). Performance of deep learning models for classifying and detecting common weeds in corn and soybean production systems. Comput. Electron. Agric. 184, 106081. doi: 10.1016/j.compag.2021.106081

[B3] Akiyama S. Dossa R. F. J. Arulkumaran K. Sujit S. Johns E. (2024). “ Open-loop vlm robot planning: An investigation of fine-tuning and prompt engineering strategies,” in First Workshop on Vision-Language Models for Navigation and Manipulation at ICRA 2024.

[B4] Alayrac J.-B. Donahue J. Luc P. Miech A. Barr I. Hasson Y. . (2022). Flamingo: a visual language model for few-shot learning. Adv. Neural Inf. Process. Syst. 35, 23716–23736.

[B5] Alharam A. Otrok H. Elmedany W. Bakht A. B. Alkaabi N. (2021). “ Ai-based anomaly and data posing classification in mobile crowd sensing,” in 2021 International Conference on Innovation and Intelligence for Informatics, Computing, and Technologies (3ICT). 225–229 ( IEEE).

[B6] Anderson K. Gaston K. J. (2013). Lightweight unmanned aerial vehicles will revolutionize spatial ecology. Front. Ecol. Environ. 11, 138–146. doi: 10.1890/120150

[B7] Asad M. H. Bais A. (2020). Weed detection in canola fields using maximum likelihood classification and deep convolutional neural network. Inf. Process. Agric. 7, 535–545. doi: 10.1016/j.inpa.2019.12.002

[B8] Awais M. Alharthi A. H. S. A. Kumar A. Cholakkal H. Anwer R. M. (2025). “ Agrogpt: Efficient agricultural vision-language model with expert tuning,” in 2025 IEEE/CVF Winter Conference on Applications of Computer Vision (WACV). 5687–5696 ( IEEE).

[B9] Bauer M. V. Marx C. Bauer F. V. Flury D. M. Ripken T. Streit B. (2020). Thermal weed control technologies for conservation agriculture—a review. Weed Res. 60, 241–250. doi: 10.1111/wre.12418

[B10] Chen Z. Cai Y. Liu Y. Liang Z. Chen H. Ma R. . (2025). Towards end-to-end rice row detection in paddy fields exploiting two-pathway instance segmentation. Comput. Electron. Agric. 231, 109963. doi: 10.1016/j.compag.2025.109963

[B11] Deng J. Liu S. Chen H. Chang Y. Yu Y. Ma W. . (2025). A precise method for identifying 3d circles in freeform surface point clouds. IEEE Trans. Instrumentation Measurement.

[B12] Din M. U. Akram W. Bakht A. B. Dong Y. Hussain I. (2025). “ Maritime mission planning for unmanned surface vessel using large language model,” in 2025 IEEE International Conference on Simulation, Modeling, and Programming for Autonomous Robots (SIMPAR). 1–6 ( IEEE).

[B13] Dubey A. Jauhri A. Pandey A. Kadian A. Al-Dahle A. Letman A. . (2024). The llama 3 herd of models. arXiv.

[B14] Esposito M. Crimaldi M. Cirillo V. Sarghini F. Maggio A. (2021). Drone and sensor technology for sustainable weed management: A review. Chem. Biol. Technol. Agric. 8, 18. doi: 10.1186/s40538-021-00217-8

[B15] Gao J. Sarkar B. Xia F. Xiao T. Wu J. Ichter B. . (2024). “ Physically grounded vision-language models for robotic manipulation,” in 2024 IEEE International Conference on Robotics and Automation (ICRA). 12462–12469 ( IEEE).

[B16] Gao X. Gao J. Qureshi W. A. (2025). Applications, trends, and challenges of precision weed control technologies based on deep learning and machine vision. Agronomy 15, 1954. doi: 10.3390/agronomy15081954

[B17] Gašparović M. Zrinjski M. Barković Radočaj D. (2020). An automatic method for weed mapping in oat fields based on uav imagery. Comput. Electron. Agric. 173, 105385. doi: 10.1016/j.compag.2020.105385

[B18] Gauba A. Pi I. Man Y. Pang Z. Adve V. S. Wang Y.-X. (2025). Agmmu: A comprehensive agricultural multimodal understanding and reasoning benchmark. arXiv.

[B19] Gazoulis I. Kanatas P. Antonopoulos N. (2021). Cultural practices and mechanical weed control for the management of a low-diversity weed community in spinach. Diversity 13, 616. doi: 10.3390/d13120616

[B20] Genze N. Wirth M. Schreiner C. Ajekwe R. Grieb M. Grimm D. G. (2023). Improved weed segmentation in uav imagery of sorghum fields with a combined deblurring segmentation model. Plant Methods 19, 87. doi: 10.1186/s13007-023-01060-8, PMID: 37608384 PMC10463442

[B21] Goel D. Kapur B. Vuppuluri P. P. (2024). Machine learning interventions for weed detection using multispectral imagery and unmanned aerial vehicles–a systematic review. arXiv.

[B22] Guebsi R. Mami S. Chokmani K. (2024). Drones in precision agriculture: A comprehensive review of applications, technologies, and challenges. Drones 8, 686. doi: 10.3390/drones8110686

[B23] Guo Z. Xue Y. Wang C. Geng Y. Lu R. Li H. . (2025). Efficient weed segmentation in maize fields: A semi-supervised approach for precision weed management with reduced annotation overhead. Comput. Electron. Agric. 229, 109707. doi: 10.1016/j.compag.2024.109707

[B24] Hasan A. M. Sohel F. Diepeveen D. Laga H. Jones M. G. (2021). A survey of deep learning techniques for weed detection from images. Comput. Electron. Agric. 184, 106067. doi: 10.1016/j.compag.2021.106067

[B25] Hu C. Thomasson J. A. Bagavathiannan M. V. (2021). A powerful image synthesis and semi-supervised learning pipeline for site-specific weed detection. Comput. Electron. Agric. 190, 106423. doi: 10.1016/j.compag.2021.106423

[B26] Huang Y. Reddy K. N. Fletcher R. S. Pennington D. (2018). Uav low-altitude remote sensing for precision weed management. Weed Technol. 32, 2–6. doi: 10.1017/wet.2017.89

[B27] Hurst A. Lerer A. Goucher A. P. Perelman A. Ramesh A. Clark A. . (2024). Gpt-4o system card. arXiv preprint arXiv:2410.21276.

[B28] Jin X. Bagavathiannan M. Maity A. Chen Y. Yu J. (2022). Deep learning for detecting herbicide weed control spectrum in turfgrass. Plant Methods 18, 94. doi: 10.1186/s13007-022-00929-4, PMID: 35879797 PMC9310453

[B29] Kuckreja K. Danish M. S. Naseer M. Das A. Khan S. Khan F. S. (2024). “ Geochat: Grounded large vision-language model for remote sensing,” in Proceedings of the IEEE/CVF Conference on Computer Vision and Pattern Recognition. 27831–27840.

[B30] Li M. Jia T. Wang H. Ma B. Lu H. Lin S. . (2024a). Ao-detr: Anti-overlapping detr for x-ray prohibited items detection. IEEE Trans. Neural Networks Learn. Syst. doi: 10.1109/TNNLS.2024.3487833, PMID: 39504297

[B31] Li L. Li J. Chen D. Pu L. Yao H. Huang Y. (2025). Vllfl: A vision-language model based lightweight federated learning framework for smart agriculture. arXiv.

[B32] Li J. Li D. Xiong C. Hoi S. (2022). “ Blip: Bootstrapping language-image pre-training for unified vision-language understanding and generation,” in International conference on machine learning. 12888–12900 ( PMLR).

[B33] Li Y. Xu M. Miao X. Zhou S. Qian T. (2024b). “ Prompting large language models for counterfactual generation: An empirical study,” in Proceedings of the 2024 Joint International Conference on Computational Linguistics, Language Resources and Evaluation (LREC-COLING 2024). 13201–13221.

[B34] Liu T. Zhao Y. Wang H. Wu W. Yang T. Zhang W. . (2024). Harnessing uavs and deep learning for accurate grass weed detection in wheat fields: a study on biomass and yield implications. Plant Methods 20, 144. doi: 10.1186/s13007-024-01272-6, PMID: 39300566 PMC11412042

[B35] Ma B. Wang Q. Xue B. Hou Z. Jiang Y. Cai W. (2022). Application of uav remote sensing in monitoring water use efficiency and biomass of cotton plants adjacent to shelterbelt. Front. Plant Sci. 13, 894172. doi: 10.3389/fpls.2022.894172, PMID: 35783946 PMC9244790

[B36] Maes W. H. Steppe K. (2019). Perspectives for remote sensing with unmanned aerial vehicles in precision agriculture. Trends Plant Sci. 24, 152–164. doi: 10.1016/j.tplants.2018.11.007, PMID: 30558964

[B37] Min X. Ye Y. Xiong S. Chen X. (2025). Computer vision meets generative models in agriculture: Technological advances, challenges and opportunities. Appl. Sci. 15, 7663. doi: 10.3390/app15147663

[B38] Nasir M. F. Fuentes A. Han S. Liu J. Jeong Y. Yoon S. . (2025). Multi-camera fusion and bird-eye view location mapping for deep learning-based cattle behavior monitoring. Artif. Intell. Agric. doi: 10.1016/j.aiia.2025.06.001

[B39] Pan J. Zhong R. Xia F. Huang J. Zhu L. Yang Y. . (2025). Chatleafdisease: a chain-of-thought prompting approach for crop disease classification using large language models. Plant Phenomics, 100094. doi: 10.1016/j.plaphe.2025.100094, PMID: 41416205 PMC12709970

[B40] Qu H.-R. Su W.-H. (2024). Deep learning-based weed–crop recognition for smart agricultural equipment: A review. Agronomy 14, 363. doi: 10.3390/agronomy14020363

[B41] Radford A. Kim J. W. Hallacy C. Ramesh A. Goh G. Agarwal S. . (2021). “ Learning transferable visual models from natural language supervision,” in International conference on machine learning. 8748–8763 ( PmLR).

[B42] Rai N. Zhang Y. Villamil M. Howatt K. Ostlie M. Sun X. (2024). Agricultural weed identification in images and videos by integrating optimized deep learning architecture on an edge computing technology. Comput. Electron. Agric. 216, 108442. doi: 10.1016/j.compag.2023.108442

[B43] Ramirez W. Achanccaray P. Mendoza L. Pacheco M. (2020). “ Deep convolutional neural networks for weed detection in agricultural crops using optical aerial images,” in 2020 IEEE latin american GRSS & ISPRS remote sensing conference (LAGIRS) ( IEEE), 133–137.

[B44] Rehman M. U. Eesaar H. Abbas Z. Seneviratne L. Hussain I. Chong K. T. (2024). Advanced drone-based weed detection using feature-enriched deep learning approach. Knowledge-Based Syst. 305, 112655. doi: 10.1016/j.knosys.2024.112655

[B45] Rodrigo M. Oturan N. Oturan M. A. (2014). Electrochemically assisted remediation of pesticides in soils and water: a review. Chem. Rev. 114, 8720–8745. doi: 10.1021/cr500077e, PMID: 24983494

[B46] Russo C. Cirillo V. Esposito M. Lentini M. Pollaro N. Maggio A. (2024). Convolutional neural network for the early identification of weeds: A technological support to biodiversity and yield losses mitigation. Smart Agric. Technol. 9, 100594. doi: 10.1016/j.atech.2024.100594

[B47] Saleem M. H. Potgieter J. Arif K. M. (2022). Weed detection by faster rcnn model: An enhanced anchor box approach. Agronomy 12, 1580. doi: 10.3390/agronomy12071580

[B48] Sarvakar K. Thakkar M. (2024). “ Different vegetation indices measurement using computer vision,” in Applications of computer vision and drone technology in agriculture 4.0. 133–163 ( Springer).

[B49] Shams M. Y. Gamel S. A. Talaat F. M. (2024). Enhancing crop recommendation systems with explainable artificial intelligence: a study on agricultural decision-making. Neural Computing Appl. 36, 5695–5714. doi: 10.1007/s00521-023-09391-2

[B50] Shen X. Li L. Ma Y. Xu S. Liu J. Yang Z. . (2025). Vlcim: A vision-language cyclic interaction model for industrial defect detection. IEEE Trans. Instrumentation Measurement. doi: 10.1109/TIM.2025.3583364

[B51] Shi D. Zhang W. Yang J. Huang S. Chen X. Xu P. . (2025). A multimodal visual–language foundation model for computational ophthalmology. NPJ Digital Med. 8, 381. doi: 10.1038/s41746-025-01772-2, PMID: 40542189 PMC12181238

[B52] Shinn N. Cassano F. Gopinath A. Narasimhan K. Yao S. (2023). Reflexion: Language agents with verbal reinforcement learning. Adv. Neural Inf. Process. Syst. 36, 8634–8652.

[B53] Sishodia R. P. Ray R. L. Singh S. K. (2020). Applications of remote sensing in precision agriculture: A review. Remote Sens. 12, 3136. doi: 10.3390/rs12193136

[B54] Stanley K. Entz M. H. (2022). New tools for mechanical weed control in low-input dry bean (phaseolus vulgaris) production. Can. J. Plant Sci. 102, 1057–1060. doi: 10.1139/cjps-2021-0282

[B55] Suzer M. H. Şenbayram M. Çullu M. A. (2024). Sustainable farming through precision agriculture: Enhancing nitrogen use and weed management. Precis. Agriculture-Emerging Technol.

[B56] Tan L. (2025). Causally-informed instance-wise feature selection for explaining visual classifiers. Entropy 27, 814. doi: 10.3390/e27080814, PMID: 40870286 PMC12385936

[B57] Team G. Georgiev P. Lei V. I. Burnell R. Bai L. Gulati A. . (2024). Gemini 1.5: Unlocking multimodal understanding across millions of tokens of context. arXiv.

[B58] Toscano F. Fiorentino C. Capece N. Erra U. Travascia D. Scopa A. . (2024). Unmanned aerial vehicle for precision agriculture: A review. IEEE Access 12, 69188–69205. doi: 10.1109/ACCESS.2024.3401018

[B59] Tsouros D. C. Bibi S. Sarigiannidis P. G. (2019). A review on uav-based applications for precision agriculture. Information 10, 349. doi: 10.3390/info10110349

[B60] Villette S. Maillot T. Guillemin J. P. Douzals J.-P. (2021). Simulation-aided study of herbicide patch spraying: influence of spraying features and weed spatial distributions. Comput. Electron. Agric. 182, 105981. doi: 10.1016/j.compag.2020.105981

[B61] Wang J. Ming Y. Shi Z. Vineet V. Wang X. Li S. . (2024). Is a picture worth a thousand words? delving into spatial reasoning for vision language models. Adv. Neural Inf. Process. Syst. 37, 75392–75421.

[B62] Wang X. Song X. Li Z. Wang H. (2025b). Yolo-dbs: Efficient target detection in complex underwater scene images based on improved yolov8. J. Ocean Univ. China 24, 979–992. doi: 10.1007/s11802-025-6029-2

[B63] Wang X. Wei J. Schuurmans D. Le Q. Chi E. Narang S. . (2022). Self-consistency improves chain of thought reasoning in language models. arXiv.

[B64] Wang A. Zhang W. Wei X. (2019). A review on weed detection using ground-based machine vision and image processing techniques. Comput. Electron. Agric. 158, 226–240. doi: 10.1016/j.compag.2019.02.005

[B65] Wang D. Zhao M. Li Z. Xu S. Wu X. Ma X. . (2025a). A survey of unmanned aerial vehicles and deep learning in precision agriculture. Eur. J. Agron. 164, 127477. doi: 10.1016/j.eja.2024.127477

[B66] Weng X. Pang C. Xia G.-S. (2025). Vision-language modeling meets remote sensing: Models, datasets, and perspectives. IEEE Geosci. Remote Sens. Magazine. doi: 10.1109/MGRS.2025.3572702

[B67] Xu H. Han F. Zhou W. Liu Y. Ding F. Zhu J. (2024). Esmnet: An enhanced yolov7-based approach to detect surface defects in precision metal workpieces. Measurement 235, 114970. doi: 10.1016/j.measurement.2024.114970

[B68] Yang X. Chen J. Lu X. Liu H. Liu Y. Bai X. . (2025). Advances in uav remote sensing for monitoring crop water and nutrient status: Modeling methods, influencing factors, and challenges. Plants 14, 2544. doi: 10.3390/plants14162544, PMID: 40872167 PMC12389011

[B69] Yang X. Wang J. Xia X. Zhang Z. He J. Nong B. . (2021). Osttg1, a wd40 repeat gene, regulates anthocyanin biosynthesis in rice. Plant J. 107, 198–214. doi: 10.1111/tpj.15285, PMID: 33884679

[B70] Yao S. Yu D. Zhao J. Shafran I. Griffiths T. Cao Y. . (2023). Tree of thoughts: Deliberate problem solving with large language models. Adv. Neural Inf. Process. Syst. 36, 11809–11822.

[B71] Yu G.-H. Anh L. H. Vu D. T. Lee J. Rahman Z. U. Lee H.-Z. . (2025). Vl-paw: A vision–language dataset for pear, apple and weed. Electronics 14, 2087. doi: 10.3390/electronics14102087

[B72] Yue X. Ni Y. Zhang K. Zheng T. Liu R. Zhang G. . (2024). Mmmu: A massive multi-discipline multimodal understanding and reasoning benchmark for expert agi. Proc. IEEE/CVF Conf. Comput. Vision Pattern Recognit., 9556–9567. doi: 10.1109/CVPR52733.2024.00913

[B73] Zhang J. Huang J. Jin S. Lu S. (2024). Vision-language models for vision tasks: A survey. IEEE Trans. Pattern Anal. Mach. Intell. 46, 5625–5644. doi: 10.1109/TPAMI.2024.3369699, PMID: 38408000

